# Do unions contribute to creative destruction?

**DOI:** 10.1371/journal.pone.0261212

**Published:** 2021-12-13

**Authors:** Harald Dale-Olsen

**Affiliations:** Institute for Social Research, Oslo, Norway; TED University, TURKEY

## Abstract

We apply a shift-share approach and historical unionisation data from 1918 to study the impact of regional unionisation changes in Norway on regional wage and productivity growth, job-creation and -destruction and social security uptake during the period 2003–2012. As unionisation increases, wages grow. Lay-offs through plant closures and shrinking workplaces increase, causing higher retirement rates, while job creation, plant entry and other social security uptakes are unaffected. Productivity grows, partly by enhanced productivity among surviving and new firms and partly by less productive firms forced to close due to increased labour costs. Thus, unions promote creative destruction.

## Introduction

In many modern economies, union membership is on the decline and has been for several years. This is seen in major industrial countries such as the UK and Germany, but also in the previously strongly organised Nordic countries [1–4]. OECD [4] reports an average decline from 33 percent unionisation in the mid-80s to 17 percent today. This is worrisome, since OECD [5] argues that unions and collective bargaining could potentially play a central role in creating *more* and better jobs, labour market inclusiveness, and resilience and adaptability. In addition, Barth et al. [6], utilising a Norwegian tax reform affecting the price of union membership to draw causal inference, find that as firm union density increases, both firm productivity and average firm wages grow, but productivity more so. Union decline could then entail productivity loss and diminishing wages and profits, and thereby have negative consequences for employers and workers.

In our study, we ask how unions influence the creation of new jobs and the destruction of old jobs, potentially affecting social security uptake. To identify the impacts of unions on job creation and destruction are, unfortunately, far from easy. The main obstacle is the absence of exogenous variation in the unionisation required to draw causal inferences. The incentives to unionise might depend on the business situation. Worker selection by abilities might also affect performance, and thereby, job creation and destruction. Such processes affect the empirical relationships between unionisation and different outcomes at most levels; across workers, across firms or across regions.

Furthermore, job creation and destruction influence economic growth through creative destruction, i.e., the process where obsolete jobs close to free resources for new productive jobs. Job destruction and creation follow from decisions made by employers and owners taking into account current and future profits. Wages and productivity are key factors in this process. To understand how unions shape job creation and destruction, one needs to study the impacts on wages and productivity as well. Thus, our study also ask if unionisation affects productivity and wages, do these impacts vary across the productivity and wage distribution? Do unions contribute to the process of creative destruction? If so, do they also affect the uptake of social security? Following plant closures and job displacements in Norway, several authors have identified inflow to welfare recipiency, either as short-term unemployment insurance or as long-term disability insurance and permanent withdrawal [7–9]. Thus, creative destruction might have long-run detrimental impact on workers.

In this paper, we utilise regional differences in Norway to study the impact of unionisation on wages, on job creation and destruction, on productivity, and on social security uptake. Our study supplements Barth et al. [6] by focusing on other aspects than productivity and wages. It also takes into account spill-over effects on the regional level, whereas Barth et al. only focused on firm-level outcomes. Furthermore, while Barth et al. focused on rent-sharing, we address the process of creative destruction. To solve the problems of drawing causal inference on the impacts of unionisation as discussed above, we apply a shift-share approach [10–12] by using information on the historical distribution of unionisation across industries.

The wage-setting in Norway is defined by Oecd [5] as *Organised decentralised and Co-ordinated*. Sector-level agreements are important, with coordination across sectors and bargaining units, but with room for lower-level agreements. One can trace this regime back to the early 20^th^ century. In our case, we use historical data from 1918. We chose this year for our historical data for two reasons. First, in the Norwegian database on historical union data, there is a gap from 1918 to the late 1960s. By focussing on pre-WWII-union data, we avoid that the local historical processes potentially affecting unions, are still ongoing today. Second, the prevalence of union members across municipalities in the pre-WWII-period is maximised by selecting the year 1918.

Our study shows that as local unionisation increases, wages grow. Lay-offs through plant closures and shrinking workplaces increase, causing increased retirement rates. Job creation, plant entry and social security uptake are unaffected. Increased local unionisation also causes productivity growth. Less productive firms close due to the increased labour costs, and thereby free resources. These resources enhance the productivities of both the surviving firms and the new entrants. Thereby, unions contribute not only to rent-sharing, but also to creative destruction.

### Previous literature

Theoretically, unions might affect both job creation and job destruction. First, there is a rich theoretical literature linking how unions bargain for wages and how this affects innovation, job creation and destruction, and employment [13–16]. In this literature, local union bargaining stifles job creation and innovations, but also reduces job destruction and firm exit. Bargaining at sector-level or at the national-level, on the other hand, yields the opposite results. Thereby unions, through higher level bargaining, are important vessels of creative destruction [17]. On the other hand, Boeri [18] argues that two-tiered wage bargaining causes allocative inefficiencies due to a decoupling of wages from productivity. Under the standard approach, the right-to-manage model ([19]), unions bargain over wages only, and the employer set the profit maximizing employment level. However, unions might bargain over other aspects than wages. Under so-called efficient bargaining [20], unions bargain for wages and employment. If unions influence effort, unions might induce efficient outcomes even under local bargaining [14]. Recently, Bryson and Dale-Olsen [21] find that local bargaining actually increases innovations in the UK and Norway.

The huge theoretical literature on how unions affect productivity yield ambiguous predictions on the relationship between unions and productivity [22–26]. Worker shirking, featherbedding and lowered capital investments are arguments associated with reduced productivity for the union firms. “Voice”-effect, capitalization and capital deepening, and improved monitoring contribute to improved productivity.

The direct empirical studies on how unions cause job creation and destruction are few and yield mixed evidence, and only DiNardo and Lee [27] provide causal inference. On U.S. data, they find no significant relationship during the period 1983–99. The correlation studies reveal that unions deter entry [28] while higher unionisation rates increase the closure rates [29, 30]. Still, a considerable heterogeneity exists (for example, over time). The empirical studies on the correlation between unionisation and employment growth report that unionisation is associated with 2–4 percent reduced employment growth [31–34], but no causal evidence is available. Still, Addison and Belfield [1] call the employment effects of unions as the “one constant”.

The empirical studies providing causal evidence on unions and productivity yield mixed evidence on productivity. The US-studies, motivated by the Wagner Act requiring majoritarian union representation at the firm, employ a RD-approach. On average, the impacts of union density on wages and productivity are small, although it appears that the size of the impact on wages actually vary across the distribution of wages [2, 9, 35], compressing wages at the top and lifting wages at the bottom. The follow-up study of Lee and Mas [36] indicates that in the long-term, the financial market’s evaluation of a firm differs if unions present at the firm have bargaining power, potentially making unionisation expensive. In contrast, and as pointed out in the introduction, Barth et al. [6] find on Norwegian data that as a firm’s union density increases, firm productivity and firm average wages grow, but productivity more so.

Several important empirical papers link the closures of establishments and firms, induced by creative destruction, to welfare utilisation, i.e., to the uptake of unemployment and disability insurance, and to the withdrawal from the labour market in the form of retirement. Particularly relevant is the literature on the consequences of job displacement. For over 30 years, numerous studies have identified negative income effects associated with job displacement [8, 37, 38], while later studies have identified additional detrimental health effects associated with the displacement process [39–42]. However, one should note that in a neighbouring Scandinavian country to Norway, Denmark, Roulet [43] observed that such detrimental health effects could, conditional on adequate policies, be negligible or even possibly avoided. Still, since displacement is often associated with detrimental health effects, many authors find positive inflows to disability pension enrolment following job loss and plant closures [7, 9, 10, 44], although Bratsberg et al. [9] argue that part of this inflow is unemployment in disguise.

### Unionisation in Norway in the early 20^th^ century and today

The first major trade union, The Norwegian Confederacy of Trade Unions (LO), was established in 1899, with 1600 members in two union braches. In 1907, LO was part in the first comprehensive sectoral trade union agreement in Norway (governing iron- and metal- workers). During the next 20 years, LO experienced massive growth. In 1920, LO comprised close to 150000 members in over 20 unions. Most of these unions are typical manufacturing unions such as book binder union, iron- and metal workers union, meat producing workers union, paper mill worker union, typographers, and tailor and textile worker union), but also unions within construction (painters, carpenters, masons and brick workers), transport (sailors, stokers, transporters), and agriculture (peasant and forest workers). Even classical service occupations were represented (barbers). Then, after a series of less successful strikes and interventions, membership dropped.

During the 1930s, with economic turmoil in most western economies, LO continued to grow, and at the same time, the worker movement gained political power as important contributor to the Norwegian Labour Party (Arbeiderpartiet), which had been founded in 1887 (Bjørnhaug et al., 2000). In 1935, the first centralised cross-sectoral agreement between LO and the employer association was signed. When the first Labour government was established the same year, the cabinet comprised two ministers from LO. LO comprised over 350000 members when the World War II started. During the post-war years LO continued to grow, and its close link to the Labour Party ensured that it continued to be an important political organisation fighting for improved wages and working conditions. Although competing confederacies of unions were established during 1980s and 1990s, LO is still by far the largest confederacy of unions in Norway, comprising over half of all union workers in Norway, with over 900000 members employed in all sectors and industries. Although this number varies slightly over the business cycle, LO organises 35–45 percent of all workers in Norway. However, sectoral differences exist. LO is stronger in the classical manufacturing industries. Competing confederacies of unions are at least equally important in the public sectors. The development of union membership levels during the first half of the 20^th^ century was by no means unique for Norway, but is observed in many countries (see Müller [45] for German figures, and Wolman [46] and Wrigley [47] for British union figures).

Holmes and Bryson et al. [48, 49] noticed in the U.S. and the U.K. that coal mines and steel mills embedded unionization locally even after these industries were long gone. In Norway, a geographically long and narrow, sparsely populated country, a similar process occurred and unions were embedded locally. At the turn of the 19^th^ century, sawmills and other power-demanding industries were established close to Norway’s major energy source in the 19^th^ and early 20^th^ century: waterfalls and rivers. Mining towns had been established long ago, as well as cities important for export and trade (e.g., at the coastline or along waterways) such as the capital Oslo. These industries embedded unions locally, and this was enforced since unions and the labour movement of the early 20^th^ century [50] also were important providers of culture locally (organisers of library services, education, song and music, sports) [51].

Still, in 1918, more than fifty percent of the municipalities were not unionized. For example, the central parts of Norway were less unionized, since in those days this mountainous area comprised agricultural activities dominated by small farms. Today, most Norwegian municipalities are heavily unionized. Table 1 provides a brief comparison between the union distribution across municipalities in 1918 and in 2003 for 6 broad industries (see the Data Section for details). In 1918, unionisation primarily occurred in Manufacturing and Mining. All these six industries experienced a massive growth in unionisation from 1918 to 2003.

**Table 1:** Union distributions within industries 1918 and 2003.

	Agriculture, forestry, fishing	Mining	Manufac-turing	Constru-tion	Transport	Others
Panel A) Union distribution 1918						
5	0	0	0	0	0	0
25	0	0	0.15	0	0	0
50	0	0.14	0.40	0.01	0.10	0
75	0	0.60	0.46	0.06	0.22	0.005
95	0.22	0.80	0.58	0.07	0.22	0.005
Mean	0.03	0.27	0.33	0.02	0.11	0.003
Total workers	99479	4000	161709	133358	91669	291003
Panel B) Union distribution 2003						
5	0.05	0.22	0.36	0.08	0.34	0.23
25	0.13	0.49	0.48	0.26	0.54	0.29
50	0.19	0.54	0.59	0.39	0.56	0.29
75	0.26	0.63	0.69	0.48	0.62	0.37
95	0.46	0.87	0.83	0.64	0.77	0.44
Mean	0.20	0.55	0.59	0.37	0.57	0.32
Total workers	19239	19134	235691	105202	135664	683580

Percentiles and averages calculated across municipalities separately for each industry. The union distribution in 1918 is calculated from unionisation figures from 1918, but Census employment figures from 1920. See also Data Section for more details.

**Fig 1:**
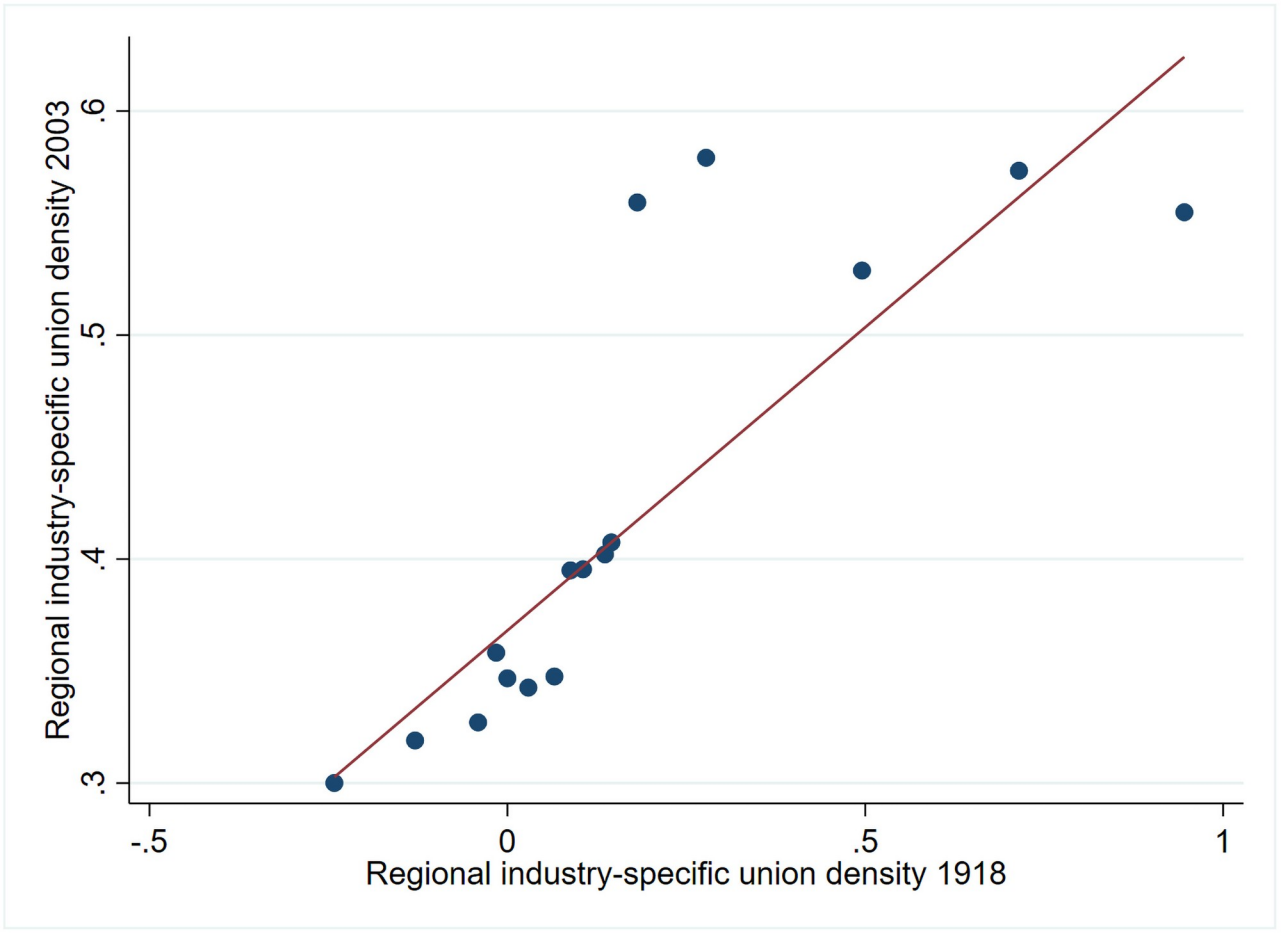
The correlation between regional industry-specific unionisation 1918 and the regional industry-specific union density 2003. Within municipality. The figures are based on averages of 20 equal-sized binned observations of the regional industry-specific union density 1918 and the regional industry-specific union density 2003. Union density in 1918 is calculated from unionisation figures from 1918 but Census employment figures from 1920. Note that one has a priori residualized these by absorbing the municipality.

Regional unionisation nearly a century ago correlates with regional unionisation today. High levels of unionisation in 1918 usually imply high levels of unionisation in 2003, and vice a versa. However, even if the regional unionisation in 1918 is related to the regional unionisation in 2003, this might hide that the underlying industry composition have changed, and thus that the unionisation within industries is not related over time. To address this worry, we residualize the regional industry-specific union density in 1918 and the regional industry-specific union density 2003 by conducting the within-transformation. Then we divide the transformed union density in 1918 into 20 equal-sized bins, and compute the means of the transformed union density 2003 within each bin. Fig 1 presents a scatterplot of these data points. Even in this rough non-parametrical example, we see a strong positive relationship within municipalities between industry-specific union density of 1918 and the union density of 2003.

### Data

The main dataset is based on public administrative register data provided by Statistics Norway comprising *all* firms, workplaces, employees and individuals in Norway 2003–2012. This data set provides information on individuals and workers (gender, educational qualifications, union membership, earnings and income, social security uptake related to unemployment, disability insurance and retirement), on jobs (occupation, seniority, spell-specific earnings and fringe benefits, working hours, wages), and on firms-and establishments/workplaces (employment, industry, sector and municipality/location). We focus on employment spells spanning December 31th each year. The social security uptake related to unemployment, disability insurance and retirement are based on receiving benefits, i.e., short spells of unemployment might thus not be registered. We measure long-term disability as receiving disability insurance still after 3 years.

The second dataset comprises the Municipality Data Base (MDB). This database comprises historical employment data and historical union membership information from the largest union in Norway: The Norwegian Confederacy of Trade Unions (LO). The first year in the MDB is 1909, 10 years after LO’s establishment. Between 1918 and to the early 1960s MNB has no information. For reasons as described in the introduction, we chose 1918 for our historical data.

The Municipality Data Base allows us to map LO union members across municipalities in 1918 at union or “branch” level. It also comprises employment data at the municipality level based on the Norwegian 1920 Census, split into six main industries. The historical data thus comprise union and worker information across municipalities and six aggregated industries.

The third dataset is the Statistics Norway’s Structural Statistics database linked to the Accounting Registers for 2001–2012. The Structural Statistics provides information on value added and industry for a majority of the private sector firms in Norway. A majority of the private-sector firms are also required to report to the Accounting registers (all limited liability firms, but not single-person firms and foundations). From this register, we get information on capital assets. From the merged data set, we then get information on key firm characteristics such as value added, capital, different kinds of costs and revenues, employment and industry-code (5-digit). Finally, we link these data by the firm-specific identifying number to our previously defined job files to get information on workplace location. These data will be used to study regional productivity. Unfortunately, this linking process leaves 30 percent of the municipalityXindustry panel units with no valid observations on productivity and capital. Small and medium-sized responsible/personal liability enterprises do not have to report to the Accounting registers. In many smaller municipalities, such firms are the only private sector businesses active.

### Auxiliary regressions

To derive our key outcome variables, we conduct a series of auxiliary regressions on job-level observations over time (wage-, hire-, quit- and social security uptake regressions) and on firm-level data over time (valued added Trans-log production function regression). The latter regression yields measures of total factor productivity (TFP). The former regressions yield residuals so human capital composition changes related to dimensions such as women, immigrant, seniority, experience and educational qualification (6 dummies) have already been taken into account when we later do our main analyses. Let us describe these auxiliary regressions briefly.

#### Job-level regressions

$$Y_{jft}=\beta_{0}+t_{t}+{\beta_{1}X}_{jft}+\tau_{jft}$$ (1)

where Y_jft_ denote different outcome variables for worker j employed at workplace f at time t. Outcomes are such as the log hourly wage, a dummy for being hired, a dummy for leaving the workplace or a dummy for next year entry on different social security benefits such as unemployment, disability (long-term disability is measured after 3 years) or retirement pension. The X-vector comprises dummies for women and immigrant, seniority (and squared), experience (and squared), and educational qualification (6 dummies). A3 and A4 Tables in S1 Appendix present the results from these regressions. These regressions are conducted for both private and public sector job level data. Let ${\hat{\tau }}_{jft}^{y}$ express the residual, where y∈w, h, q, or s indicate whether the residual is from the wage regression, the hire regression, separation regression or social security uptake regression, respectively.

#### Firm-level regression

We do not observe total factor productivity (TFP) directly in the data. To measure TFP at the regional level, we start by estimating an auxiliary regression based on the firm data motivated by a simple Trans-log production function:

$${LnVA}_{ft}=\beta_{0}+t_{t}+{\beta_{1}lnC}_{ft}+{\beta_{2}{lnC}_{ft}^{2}}_{ft}+{\beta_{3}lnL}_{ft}+\beta_{4}lnL_{ft}^{2}+\beta_{5}{{lnL}_{ft}*lnC}_{ft}+\omega_{ft}+\tau_{ft},$$ (2)

where TFP then is expressed by *ω*_*ft*_, and L and C denote workforce size and capital, respectively (and corresponding squared terms and interactions) and lnVA express value added. *τ*_*ft*_ expresses a unobserved transitory shock. While a Cobb-Douglas production function introduces the strict assumptions of constant elasticity of scale and a substitution elasticity between labour and capital equal to 1, the Trans-log production function relaxes these assumption and thus rests on flexible assumptions. The classical estimation problem associated with 2) is the *endogeneity of transitory inputs*. Important contribution providing solutions to this are Levinsohn and Petrin [52] and Wooldridge’s [53] control function approach, where one includes a proxy for time varying productivity, *ω*_*ft*_ using lagged values of capital and materials and their interactions (third order polynomial) directly in the production function. We follow the Ackerberg et al. (ACF) [54]–framework, where we let labour be determined before the intermediate inputs and the realization of the productivity shock. Unobserved productivity is assumed to follow a Markov process. The proxy-variable then depends on labour as well as labour and the state variable capital. The first stage cleanses *τ*_*ft*_ from *LnVA*_*ft*_ By exploiting the Markov chain assumption, i.e., *ω*_*ft*_ = E(*ω*_*ft*_ | *ω*_*ft*-1_) + *ξ*_*ft*_ = g(*ξ*_*ft*_) + *ξ*_*ft*_, one can derive the second stage GMM-criterion-function. This two-stage GMM-approach by Ackerberg et al. is described and discussed closer in Rovigatti and Mollesi [55] and is applied in many studies, e.g., Dale-Olsen and Finseraas [56].

A5 Table in S1 Appendix presents the results from this regression. The ACF-approach let us directly estimate the unobserved total factor productivity, ${\hat{\omega }}_{ft}^{VA}$ for each firm in the Norwegian economy for the years 2003 to 2012.

#### Key variables

Our main analysis will be conducted at the municipalityXindustry-level, and we follow these units over time. Let mi and t denote municipalityXindustry and year, respectively. Note t ∈ 2003–2012. Let l_f(mi)t_ and u_f(mi)t_ denote the number of workers and the number of union workers employed at workplace f (in municipalityXindustry) at time t. Let the regional average yearly unemployment rate, which act as a business cycle control, be denoted as UN_mt_.

Our key outcome variables are then the municipalityXindustryXyear averages of the residuals from the auxiliary regressions described in the previous sub-section, for wages, job entry, job separations, inflow to unemployment, short- and long-term disability and retirement. The auxiliary production function regression yielded an estimate of firm-specific TFP. Formally, our key outcome variables can be described as:

1. Regional industry-specific average of different kinds of $\text{residuals}=(\frac{\sum_{ft}{\hat{\tau }}_{f\left(mi\right)t}^{k}}{\sum_{ft}l_{f\left(mi\right)t}})$ if f ∈ private sector, and k ∈ w, h, q, s.
  These residuals could be from log hourly wage regressions, hiring regressions (for entry jobs or job creation), separation regressions (for exit jobs or job destruction) or from inflow to social security schemes regressions (e.g., unemployment, short-term disability, long-term disability and retirement). Inflow to long-term disability by those employed at year t by firm f in municipalityXindustry mi is measured by year t+3. For wages, we also calculate the wage dispersion (standard deviation of the residuals, and the 5^th^ and 95^th^ percentile of the wage distribution within the panel unit).
2. Regional industry-specific total factor productivity: $TFP_{\text{mit}}=(\frac{\sum_{ft}{\hat{\omega }}_{f\left(mi\right)t}^{}}{\sum_{ft}F_{f\left(mi\right)t}})$ if f ∈ private sector, and F denotes the number of firms located in municipality m and industry i at time t.
  For TFP, we also calculate the 5^th^ and 95^th^ percentile of the productivity distribution within the panel unit, and the productivity dispersion (as the difference between 95^th^ percentile and the 5^th^ percentile). Using the same formula as described above, but replacing TFP with log fixed assets and log workforce size, we calculate the same regional measures for log fixed assets and log workforce size.

Finally, we identify entry firms and exit firms, and separately for entry and exit firms, calculate the regional averages of TFP, capital and workforce size associated with the entry and exit, using observations *for the entry and exit years only*. We also measure regional averages of TFP, capital and workforce size associated with *incumbent firms present all years*. When we focus on entry, on exit and to a lesser degree, on incumbents, this implies significant drops in the number of observations. A majority of the municipalitiesXindustries do not experience entry or exit of firms. The motivation for this analysis is to describe the average growth in TFP, capital and workforce size averages associated with entry, exit and incumbent firms when the regional unionisation grow, acknowledging that these analyses are estimated on highly selective samples.

### Empirical strategy

Our simple empirical strategy is based on 2-Stage Least Squares linear regressions on municipalityXindustry observations from *the private sector*, but where we first-difference the observations to take into account municipality-industry fixed effects. Our industry definition is quite broad and just based on six broad categories (agricultural, mining, manufacturing, construction, transport, others) due to the use of historical data.

Let the panel unit, municipalityXindustry, be denoted by mi. Let time be denoted by t, t ∈ 2005–2012 (following the use of the first-difference operator and the presence of lagged right-hand-side variables on data spanning 2003–12). Similarly, let m denote municipality, while i denote industry. We remove municipalityXindustry fixed effects by first-differencing the data. The empirical specification (after first-differencing) can be expressed:

$${\Delta Y}_{mit}=b_{0}+t_{t}+{b_{1}\Delta lnU}_{mit-1}+b_{2}\Delta lnL_{mit-1}+b_{3}{\Delta X}_{mt-1}+b_{4}t_{ijt}+b_{5}t_{mt}+\epsilon_{mit}$$ (3)

where Y_mit_ denotes different outcome variables such as the average log hourly wage residual, the hiring rate residual due to plant entry, the quit rate residual due to plant closure, TFP (total factor productivity), log fixed assets, log workforce size or social security uptake. Δ expresses the first-difference operator. For example, *ΔY_mit_* = *Y_mit_* − *Y_mit_*_−1_.

U_mit-1_ and L_mit-1_ denotes at time t-1 the number of union workers and the number of workers in municipalityXindustry mi, respectively.

Our key parameter of interest, *b*_1_, measures the impact of relative unionisation growth on growth in Y conditional on the relative growth in employment. When Y expresses log hourly wage, the residual wage or TFP, then *b*_1_ directly expresses an elasticity.

Note also that the terms *b*_1_Δ*lnU*_*mit*−1_ + *b*_2_Δ*lnL*_*mit*−1_ of Eq 3) can be rearranged as ${b_{1}\Delta ln\frac{U_{mit-1}}{L_{mit-1}}}_{}+a_{2}\Delta lnL_{mit-1}$, where a_2_ = b_1_+b_2_. Thus, Eq 3) yields evidence on the impact of regional union density on a regional outcome.

X_mt-1_ denotes a vector of exogenous control variables. In practice, this vector only comprises the local (municipal) unemployment rate. t_t_ and t_mt_ denote year dummies and linear municipality trends. To address linear industry trends, denoted by t_jt_, we collapse the dummies associated with 5-digit industry codes to the six industries of the panel unit and multiply these shares with a linear time trend. Finally, *ϵ*_*mit*_ denotes a classical error term.

Although Eq 3) only controls for one time-varying covariate, the municipality unemployment rate, which take into account business cycle effects, the auxiliary regressions creating the residuals (which constitute our dependent variables in Eq 3) take into account time-varying human capital covariates.

By assuming exogenous right-hand-side variables, this model could have been estimated using OLS. In such regressions, where observations constitute municipalityXindustry averages, the variance of the error terms would be diminishing by the number of observations utilised in constructing the municipalityXindustry average, thus the error-terms are heteroscedastic. To take into account this, all observations are weighted by the inverse of the number of observations within municipalityXindustry. All reported standard errors are cluster-adjusted on the panel unit.

To estimate Eq 3) by OLS is, however, not appropriate, since we cannot discard the presence of other time-varying municipalityXindustry variables causing omitted variable bias. In other words, we cannot rule out that ΔU_mit−1_ and *ϵ*_*mit*_ are correlated. Why? The classical error term in Eq 3) could express the difference of a classical error term *τ* over time, i.e., *ϵ*_*mit*_ = Δ*τ*_*mit*_ = *τ*_*mit*_ − *τ*_*mit*−1_. If the local industry-specific shock to the outcome variable in t-1, or other time-varying omitted variables affecting the outcome, could be correlated to local unionisation in the same period, then Corr (U_mit−1_, τ_*mit*−1_) ≠ 0. Such a correlation arises if bad times locally, make workers unionise to get insurance. It would also arise if something makes selected occupations more productive locally. For example, if the introduction of new infrastructure or the implementation of new technology influences the productivity of occupations differently, and these occupations happen to be unionised to a varying degree, then such a correlation arises.

To address the endogeneity and omitted variable issues raised in the introduction and above, we invoke a Bartik-like instrument following a shift-share approach [11, 12, 57]. The motivation for the shift-share instrument is to use variation in the national flows to generate variation at the local level: The expected flow to/from unionisation in an industry in a region is a weighted average of the national flows for each industry, with weights that depend on the historical distribution of unionisation across industries. We thus exploit the fact that regions are differently affected by industry variation depending on their initial industry mix. In our case, the initial mix is given by the 1918-unionisation. Our instrument is the topics for the next sub-section.

### Instrument variable

We derive our instrument by the standard shift-share strategy. Let the historical distribution of unionisation be denoted by $\frac{U_{mi0}}{U_{i0}}$, where subscript 0 denote time 0, i.e., 1918. Subscripts i and mi still denote industry and municipalityXindustry, respectively.

Following Autor and Duggan [10], we calculate the leave-one-out aggregate growth in unionisation in absolute numbers, i.e., ${\ddot{U}}_{mit-1}=\Delta \left(U_{it-1}-U_{mit-1}\right)$, where Δ expresses the first-difference operator, U_it-1_ expresses the national level of unionisation industry i and *U*_*mit*−1_ expresses the number of union workers in municipalityXindustry mi. Thus, for each observation any local contribution is cleansed from the national growth in unionisation in absolute numbers.

Our instrument is expressed as:

$$\Delta \tilde{U_{mit-1}}=\Delta {\ddot{U}}_{mit-1}\left(\frac{U_{mi0}}{U_{i0}}\right).$$ (4)

Thus, we use the historical distribution of unionisation across municipalities and industries to define the shares of the aggregate unionisation flows today, thereby predicting the growth in regional industry-specific unionisation. In other words, we utilise a shock in unionisation at the aggregate national level for these six industries, and use the historical weights to create variability.

To avoid size effects, we include growth in log number of all workers in all future regressions. Otherwise, one might worry that our instrument just picks up a mechanical relationship: large cells have more union workers just by being large, and when true for today, it would also be true for 1918. This is not true when conditioned on size. No evidence supporting this notion, is found in levels, nor is it obvious that the variance in unionisation is larger in large cells than small cells, when conditioned on employment changes.

**Fig 2:**
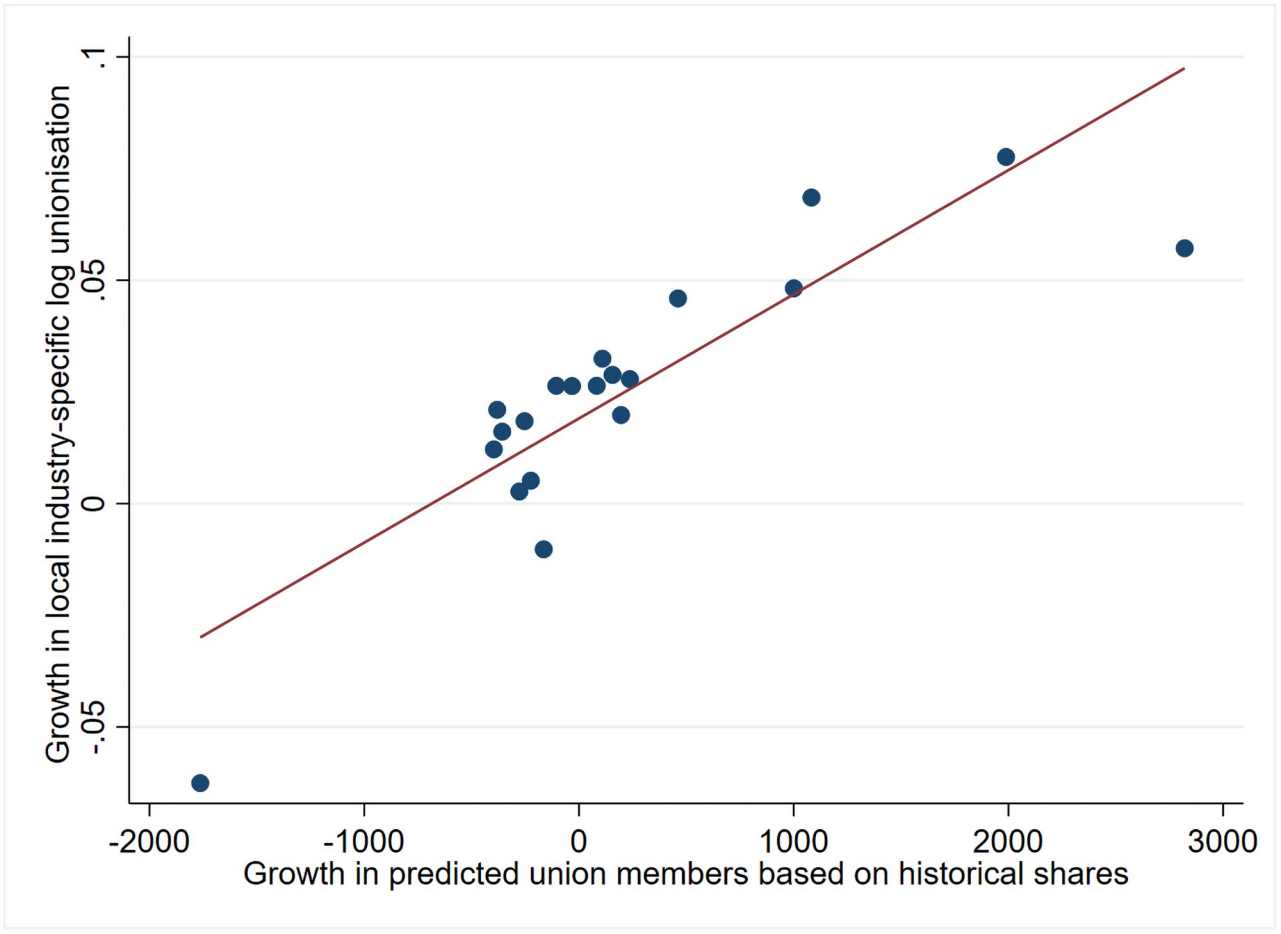
The correlation between changes in regional industry-specific unionisation and changes in the predicted numbers of regional industry-specific union members. The figure is based on averages of 20 equal-sized binned observations of the change in regional industry-specific log unionization and the numbers of union members predicted from historical shares and aggregate union members figures from the period 2003–2012. Note that one has a priori first-differenced data and then residualized these by applying a regression controlling for year dummies, lagged regional unemployment and lagged log workforce.

How does the predicted growth in regional industry-specific unionisation (our instrument) relates to growth in log regional industry-specific observed unionisation? In Fig 2, we have divided the changes in predicted local number of union members within each industry into 20 equal-sized bins, computed the means of the predicted union member change and the changes in log observed unionization within each bin, and created a scatterplot of these data points. In this rough non-parametrical example, we see a strong positive relationship between the growth in the predicted number of union members and the percentage growth in observed union members.

We use growth in lagged predicted unionisation as an instrument for the growth in log number of unionised workers in a set of IV-regressions based on first-differenced data, where the panel unit is municipalityXindustry. Since the historical shares are fixed, identification is then ensured by variation in the treatment intensity over time (where aggregate industry variation in unionisation induces the variation in local treatment intensity). While our instrument is based on standard procedures in the shift-share literature, standard shift-share approach would be to conduct the regressions at the municipality-level. By doing the analyses on first-differenced data, with municipalityXindustry as the panel unit, we effectively control for all fixed municipalityXindustry characteristics. As we will show later, our instrument remains strong.

By applying this approach, one avoids that correlations between local unionisation and local industry-specific shocks to the outcome variable, contaminate and bias the regressions. This comes at a cost. We need to assume heterogeneous treatment effects and that our IV is capturing a local average treatment effect. Our analyses compare municipalityXindustries historically heavily unionised to those less unionised or without any historical unionisation. For the municipalities with no historical unionisation, our instrument does not vary over time, i.e., they will not contribute to the identification. This means that our IV-analyses provide no information on the municipalities with no historical unionisation, but these municipalities employ a minority of the workers.

In A1 Table of S1 Appendix, we present descriptive statistics across our period of observation on key variables in the regressions. In A2 Table of S1 Appendix, we present yearly descriptive statistics on lagged unionisation, lagged predicted unionisation and lagged employment. We have split the data in three categories depending on growth in the lagged predicted number of unionised workers. Mid-column shows the group of panel units (municipalityXindustry) which do not experience any growth in the lagged predicted unionisation. These units were not unionised historically, which again implies that they get assigned the value of zero. In the regression analyses later, we discard observations from municipalities not historically unionised and repeat the regression as one of the robustness checks.

In A2 Table of S1 Appendix, Panel A) presents descriptives (mean/standard deviation) on growth in lagged unionisation, growth in lagged predicted unionisation (our instrument) and lagged employment growth. Panel B) presents the yearly aggregate sums of lagged unionisation, lagged predicted unionisation and lagged employment. A2 Table in S1 Appendix reveals large variation over time, both for the variables measured in growth and in levels. In Panel A), we see that unionisation and predicted unionisation correlate positively across the years. Even under the financial crisis considerable growth occurred. Panel B) shows that although many workers are employed in panel units not historically unionised, over half of the private sector workforce is employed in panel units unionised historically.

## Results

### Wages

We start by focussing on the regional-industrial wage regressions. Our purpose is to reveal the impact of unionisation growth on wage growth. Table 2 presents the results from these IV-regressions. Although we expect the OLS-regression results to be biased, since local inflow to unionisation will be related to local unobserved economic conditions, we present the results from the corresponding OLS-regressions in the Appendix (A6 Table in S1 Appendix) for completeness. The dependent variables are municipal-industry average of the wage residuals from individual regressions controlling for human capital variables (women, migrant, years of education, experience, and seniority) and 5-digit industry. Table 2 also presents the first-stage results. We see that our instrument in the first-stage regressions are strongly significant and clearly pass the test for strong instruments.

**Table 2:** The impact of regional industry-specific unionisation growth on growth in industry-specific regional log hourly wage residuals. First-difference linear IV-regressions.

IV				Historically unionised municipalities only	Union	Non-union
	Model 1	Model 2	Model 3	Model 4	Model 5	Model 6
**IV 2.step**						
ΔLagged lnU	0.043**	0.080**	0.034**	0.063**	0.122**	0.047*
	(0.002)	(0.017)	(0.008)	(0.018)	(0.022)	(0.020)
ΔLagged lnL	-0.054**	-0.103**		-0.084**	-0.158**	-0.061**
	(0.002)	(0.023)		(0.024)	(0.023)	(0.023)
ΔLagged lnL manu-facturing			-0.009*			
			(0.003)			
*Controls*						
Basic	Yes	Yes	Yes	Yes	Yes	Yes
Linear trends		Yes	Yes	Yes	Yes	Yes
**IV 1. step**						
$\tilde{\Delta U}$ (in 1000)	0.013**	0.014**	0.030**	0.013**	0.015**	0.013**
	(0.001)	(0.001)	(0.001)	(0.001)	(0.001)	(0.001)
ΔLagged lnL	1.273**	1.286**	0.107**	1.294**	1.234**	1.336**
	(0.042)	(0.045)	(0.028)	(0.065)	(0.045)	(0.045)
*Strength*						
F-value	158.32	140.90	158.17	85.57	142.28	114.61
MxIxT	15284	15284	15284	7148	14793	15218

Panel unit: municipalityXindustry. Population yearly municipalityXindustry-sum and averages based on *all private sector* jobs, except models 4, 5 and 6, which repeat the analyses for municipalities historically unionised, and for union and non-union workers separately. Dependent variable (Y): log hourly wage residual (see A3 Table in S1 Appendix). Control vector: Basic = lagged municipality unemployment rate, year dummies; Linear trends = linear industry trends, linear municipality trends. Each observation is weighted by the number of workers. Standard errors adjusted for panel unit-clustering reported in parentheses.

** 1 percent level of significance.

* 5 percent level of significance.

Model 1 controls for the lagged municipality unemployment rate, year dummies and lagged log employment to the control vector. In the remaining models, we also incorporate linear industry trends and linear municipality trends. Since all regressions are conducted on first-differenced observations, municipality-industry fixed effects are taken into account in all specifications. Since unionisation historically arguably comes from manufacturing, Model 3 repeats the analysis of Model 2, but controls for manufacturing employment instead of total employment (in line with Autor et al. [12]). In Model 4, we discard all observations from municipalities not historically unionised. As pointed out previously and seen in Table 1, close to 50 percent of the municipalities were not unionised in 1918, thus this has dramatic impact on the number of observations. In models 5–6, we repeat these analyses of Model 2 but let the municipal-industry wages be averaged for union and non-union workers separately.

The 2^nd^ stage results reveal that increased unionisation implies higher wages, typically yielding wage elasticity estimates of 0.04–0.08, i.e., if local unionisation increases by 1 percent, then wages grow by 0.04–0.08 percent. Secondly, controlling for manufacturing employment reduces the estimated impact, but it is still considerable and significant. Thirdly, discarding municipalities not historically unionised does not changes our results qualitatively. Finally, we see that increased regional unionisation raises wages most strongly for unionised workers, but even the wages of non-union workers increase as well. Thus, the bargaining efforts provided by unions in raising union wages, actually also benefit non-union workers, i.e., increased unionisation raises the wage level locally for all employed workers. However, the increased wage level might induce reduced labour demand and thus be costly for workers since potentially the risk of unemployment increases.

#### Additional robustness checks

Recently the shift-share approach has been criticized for not being able to eliminate the bias arising in the OLS-regressions, partly for not recognising the different sources of bias and partly for letting the identification rest on an assumption the industry shares are exogenous [58, 59]. Potentially, these concerns cause problems, but we think they are less problematic in our case.

First, our industry shares are measured close to 100 years earlier, thus most direct labour supply and demand responses related to local industry shocks in the early 20^th^ century should have died out many years ago, and since they are fixed, no worries regarding serial correlation arises.

Second, we control for fixed industryXmunicipality effects. This eliminates bias caused by permanent productivity differentials between these industries within municipalities. If unionisation varies consistently between high and low productivity industries, even within municipalities, this will not influence our estimates.

Third, we are less concerned for bias due to labour supply shocks. Our main specifications analyse wage- and hiring-/separation-residuals, cleansed for human capital characteristics (women, immigrant, education, seniority, experience) as well as 5-digit industry characteristics. Goldsmith-Pinkham et al. [59] suggest a study of the correlations between the industry shares and the labour market characteristics at the first observation year in the analysis. Thus, we have used the historical union industry shares and conducted a principal component analysis of the matrix of the 1918 industry shares across municipalities (based on the pca-procedure of STATA), and predicted the first principal component. The first principal component of the matrix of the 1918 industry shares across municipalities is mostly uncorrelated to most other local human capital characteristics of 2003 (see A9 Table in S1 Appendix). The exception is municipality size, but most regressions control for size differentials.

Fourth, following Jaeger et al. [58] and addressing worries regarding different short- and long-run impacts, we add twice lagged variables (and instruments). As seen in A10 Table of S1 Appendix, we lose a few thousand observations, but we still find positive effects from lagged unionisation growth on wages. The inclusion of log unionisation lagged two periods does not change the estimate associated with lagged unionisation growth qualitatively. Furthermore, (growth in) log unionisation lagged two periods does not have any significant impact on wages.

Fifth, we have cleansed the wage growth from what we would predict based on our instrument, and then examined how this residualised wage growth influences future values of our instrument. This yields a positive, but non-significant correlation (table not shown).

Sixth, we have tested for over-identification following Goldsmith-Pinkham et al. [59]. We have constructed an IV-vector comprising the first principal component of the matrix of the 1918 industry shares across municipalities multiplied by the year dummies. It is these interactions (the varying impact of the union industry shares over time), that ensure identification. Then we have re-estimated the model. In A11 Table of S1 Appendix, we see that this instrument vector remains strong and passes the over-identification test. Thus, we find no evidence indicating that the monotonicity-assumption is not satisfied. The final wage elasticity impact is 0.04, which is in line with the previous estimates, which we prefer due to the simpler construction.

### Entry and closure of plants

After having established that increased local unionisation causes local wage growth, we then turn to the issue of how this affects the entry and closure of plants locally, with emphasis on the number of jobs created or lost. We also look closer on job creation and destruction in surviving workplaces. Table 3 presents the results from IV-regressions of residualised entry-hires, closure-layoffs, workforce growth hires, and layoffs (see A7 Table in S1 Appendix for the OLS-results). As noted previously, we have already taken into account human capital and industry differences. We present one set of models, controlling for lagged unemployment rate, time trends and linear industry and municipality trends.

**Table 3:** The impact of regional unionisation growth on residualised job creation and destruction. First-difference linear IV-regressions.

	Hires		Separations	
	Entry	Job creation	Job destruction	Exit
ΔLagged lnU	-0.013	-0.005	0.033**	0.166**
	(0.021)	(0.032)	(0.011)	(0.025)
ΔLagged lnL	-0.055	-0.125**	-0.053**	-0.210**
	(0.030)	(0.052)	(0.016)	(0.036)
*Controls*				
Basic	Yes	Yes	Yes	Yes
Linear trends	Yes	Yes	Yes	Yes
MxIxT	15284	15284	15284	15284

Note: Population yearly municipalityXindustry-sums and averages based on *all private sector* jobs. Dependent variable (Y): hires due to plant entry/hires in growing plants/separations in decreasing plants/layoffs due to plant closure (residuals, see A3 Table in S1 Appendix). Control vector: Basic = lagged municipality unemployment rate, year dummies; Linear trends = linear industry trends, linear municipality trends. See Table 2 for information on first step parameter estimates and strength of instrument. Standard errors adjusted for panel unit-clustering reported in parentheses.

** 1 percent level of significance.

* 5 percent level of significance.

The IV-analyses reveal no impact of unionisation on neither entry nor job creation, but increased layoffs rates due to closures and increased separations when workplaces reduce their size. Thus, the increased wages following unionisation we found in Table 2 comes at a cost, in the form of job losses. Furthermore, the impact is much stronger for jobs lost to closures than for the job loss due to reduced labour demand of surviving firms.

The wage growth following increased unionisation thus forces some workplaces to close. Job creation, either by plant entry or by job growth in existing plants, is unaffected, i.e., the wage growth following increased unionisation does not deter the entry of plants or creation of new jobs. This implies that local employment drops and non-employment will increase.

Is this evidence of rent-sharing or creative destruction? While reduced labour demand among surviving firms following wage increases, is in accordance with rent-sharing (assuming that productivity also grow), it is hard to relate job loss due to plant closures to rent-sharing. It is tempting to interpret this as evidence supportive of the notion that the wage growth forces the least productive firms in the local market to close, i.e., that the local productivity threshold for surviving firms is lifted. Closures then free resources for new firms and for productive surviving firms. Since entry rates is unaffected, average local productivity should increase. Thus, in some ways, our findings so far are supporting the notion of creative destruction. In the next section, we look closer on how unionisation affects the productivity of firms, and relate our findings to creative destruction.

### Productivity and capital

As discussed in the Data Section, we do not have information on productivity in all the panel units (municipalitiesXindustries), and do our analyses on a subset of the data. Since the population of panel units has changed, we conduct both wage and productivity regressions. Table 4 presents the results from the wage regressions, where we only focus on the IV-regressions.

**Table 4:** The impact of regional unionisation growth on regional log hourly wage residual growth. First-difference linear IV-regressions.

	Mean	95–5	5	95
**2.step**				
ΔLagged lnU	0.102**	-0.002	0.144**	0.143**
	(0.026)	(0.072)	(0.054)	(0.069)
ΔLagged lnL	-0.136**	-0.004	-0.186*	-0.191*
	(0.040)	(0.099)	(0.073)	(0.095)
*Controls*				
Basic	Yes	Yes	Yes	Yes
Linear trends	Yes	Yes	Yes	Yes
**IV 1.step**				
ΔLagged $\tilde{U}$ (in 1000)	0.013**	0.013**	0.013**	0.013**
	(0.001)	(0.001)	(0.001)	(0.001)
ΔLagged lnL	1.338**	1.338**	1.338**	1.338**
	(0.029)	(0.029)	(0.029)	(0.029)
*Strength*				
K-P F-value	79.51	79.51	79.51	79.51
MxIxT	10449	10449	10449	10449

Panel unit: municipalityXindustry. Population wage regressions: yearly municipalityXindustry-sums and averages based on all private sector jobs. Control vector: *Basic* = lagged municipality unemployment rate, year dummies; *Detailed industry* = industry-shares of 5-digit industry codes; *Linear trends* = linear industry trends, linear municipality trends. Each observation in the wage regressions is weighted by the number of workers. The wage measures are based on the residuals from an auxiliary individual-level log wage regression (see A3 Table in S1 Appendix), where 5-digit industry dummies are already controlled for. Each observation is weighted by the number of workers. Standard errors adjusted for panel unit-clustering reported in parentheses.

** 1 percent level of significance.

* 5 percent level of significance.

^x^ 10 percent level of significance.

First, we see that running the wage regression on these observations yields similar, but slightly stronger results compared to the previous results. The IV-regression yields an union wage elasticity estimate of 11.6 percent, which is close to our estimate of 8 percent from Model 2 of Table 2. Unions raise wages equally at the bottom of the wage distribution (in our case measured by the 5-percentile) as at the top (i.e., the 95 percentile), with less impact on the wage dispersion (as measured by the 95–5 differential).

Next, in Table 5 we turn to the analyses of productivity, capital and workforce size. First, we see that unionisation growth causes regional TFP growth, i.e., if local unionisation increases by 1 percent, total factor productivity increases by 0.175 percent. Thus, increased local unionisation causes productivity to grow more than the labour costs (since the elasticity of wages w.r.t. unionisation is 0.10 as seen in Table 4 and the elasticity of value added on labour, except for extremely large firms, is less than 1 as is seen in A5 Table of S1 Appendix).

**Table 5:** The impact of regional unionisation growth on regional growth in total factor productivity, log capital and log employment. First-difference linear IV-regressions.

	Mean	95–5	5	95	Mean-Exit	Mean-Entry	Mean-incumbent
**A) 2.step Total factor productivity**							
ΔLagged lnU	0.175**	0.294*	-0.011	0.283**	0.682	3.176**	0.135**
	(0.036)	(0.142)	(0.068)	(0.139)	(0.398)	(0.658)	(0.044)
**B) 2.step Log fixed assets**							
ΔLagged lnU	0.521**	2.196**	0.233	2.429**	3.046*	9.928**	0.184
	(0.132)	(0.445)	(0.261)	(0.389)	(0.299)	(2.020)	(0.171))
**C) 2.step Log workforce size**							
ΔLagged lnU	0.100	1.106**	-0.011	1.095**	1.410	3.488**	-0.012
	(0.065)	(0.307)	(0.037)	(0.303)	(0.862)	(0.808)	(0.037)
*Controls*							
All models comprise the same set of controls: Basic+detailed industry+ linear trends.							
**1.step ΔLagged lnU**							
ΔLagged $\tilde{U}$ (in 1000)	0.027**	0.027**	0.027**	0.027**	0.024**	0.020**	0.025**
	(0.001)	(0.001)	(0.001)	(0.001)	(0.001)	(0.001)	(0.001)
**K-P F-value**	112.3	112.3	112.3	112.3	44.90	44.10	112.3
MxIxT	10449	10449	10449	10449	4091	3062	9243

Panel unit: municipalityXindustry. Population wage regressions: yearly municipalityXindustry-sums and averages based on all private sector jobs. Dependant variable denoted by column head: Panel A) Total factor productivity, Panel B) Log fixed assets, and C) log workforce size. Control vector: *Basic* = lagged municipality unemployment rate, year dummies; *Detailed industry* = industry-shares of 5-digit industry codes; *Linear trends* = linear industry trends, linear municipality trends. The TFP-estimate is based on the estimation of an auxiliary firm-level Translog production function estimation (see A5 Table in S1 Appendix). Each observation in the TFP-, log fixed assets or log workforce size regressions is weighted by the number of workplaces in municipalityXindustry. Standard errors adjusted for panel unit-clustering reported in parentheses.

** 1 percent level of significance.

* 5 percent level of significance.

^x^ 10 percent level of significance.

Second, when we study the impacts on the top and bottom of the productivity distribution and on the productivity dispersion, we find that local unionisation growth strongly induces higher productivities on average and at the top, but it does not affect the productivity at the bottom in the labour market. Thereby, local productivity dispersion also increases following unionisation growth.

Third, we see that the increased unionisation is associated with more capital-intensive firms, both on average and for the largest firms. If local unionisation increases by 1 percent, fixed assets increases by 0.5 percent on average.

Fourth, increased unionisation implies a growth in employment only for the largest firms. In this sample based on larger municipalities and firms reporting accounting information, increased local unionisation has only minor impact on firm workforce size. From Table 3, however, for all firms, we observed a decline in local employment. Thus, overall it appears that increased local unionisation shifts production towards being more capital-intensive.

Fifth, when we look closer at the regional productivity growth based on the entry and the exit firms only, we see that when local unionisation grows and causes local productivity growth, firm entry contributes significantly to this result. The productivity growth point estimate based on the observations from the exit firms, indicates growth on average as well, but we see that the standard error is huge, thereby revealing huge variation in closing firms’ productivities. Finally, we see that the average productivity growth for the incumbent firms following increased unionisation is only slightly weaker than what we found on average for all firms.

### Social security uptake

**Fig 3:**
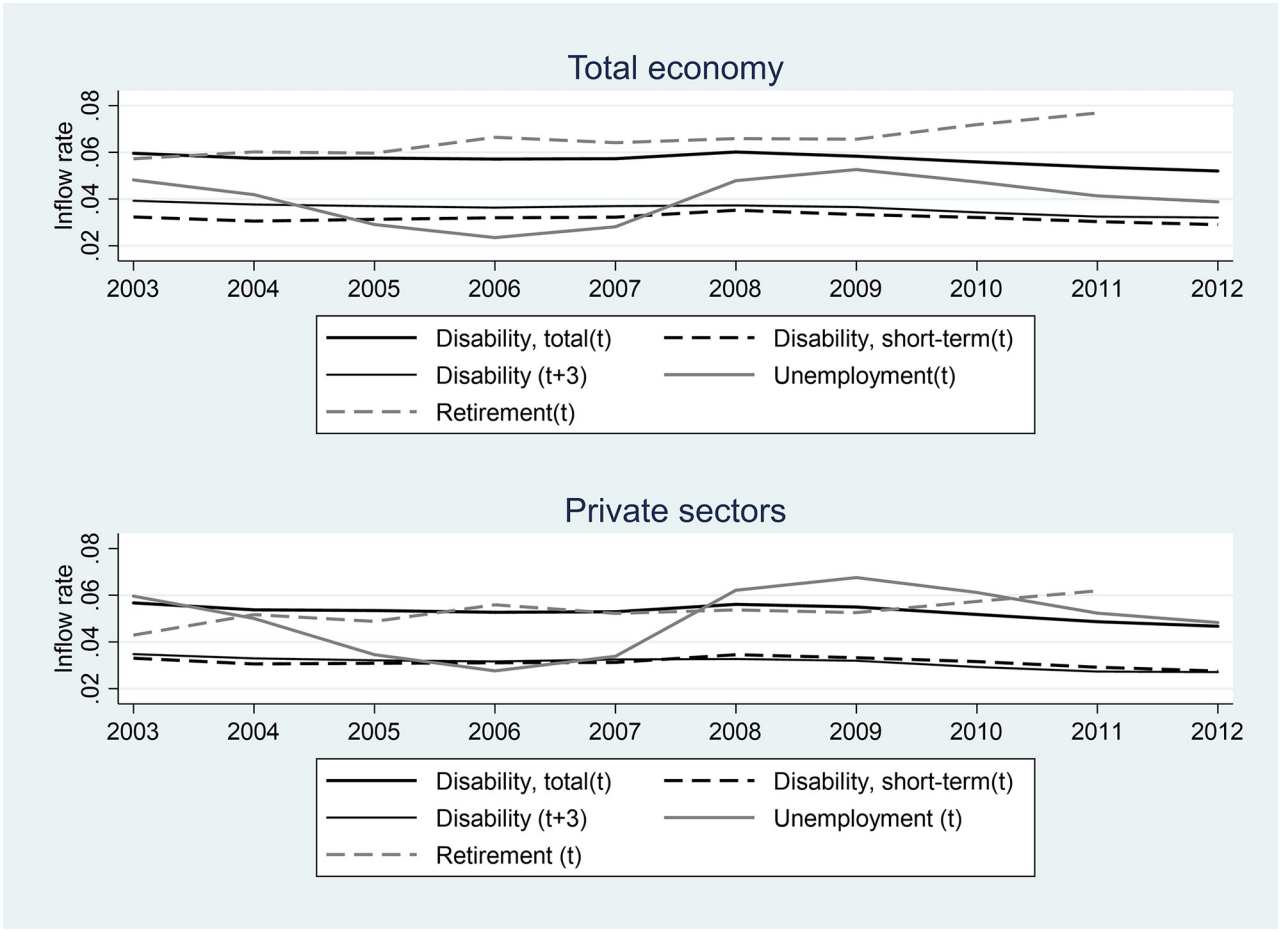
Changes in social security uptake over time. The figures are based on workers employed year t and the inflow to social security the next year (except disability-long-term which are measured after 3 years) from the period 2003–2012.

We start our empirical analyses by looking closer at the development of social security benefits uptake over our period of observation (2003–2012). Fig 3 shows the inflow to different social security schemes in year t+1 for workers employed at the end of year t. Note that these classifications rest on the receipt of benefits, very short spells might thus go unnoticed. We also measure the rate for workers receiving disability insurance still after 3 years. Top half of the figure depicts these flows for the total economy, while the bottom half shows the flows for the private sectors. Fig 3 clearly reveals the business cycle in Norway. We see that the inflow to unemployment is high 2003–4, then drops, for then again to raise during the Financial Crisis of 2008–9. Temporary short-term disablement reveals similar pattern, albeit much weaker. This indicate that bad times influence the inflow to disability and supports the notion that part of the enrolment to disability is unemployment in disguise.

Next, we turn to the issue of how these social security uptake variables relate to unionisation. Table 6 presents the results from IV-regressions of residualised social security uptake regressions (see A8 Table in S1 Appendix for the OLS-results). As noted previously, we have already taken into account human capital and industry differences. We present one set of models, controlling for lagged unemployment rate, time trends and linear industry and municipality trends.

**Table 6:** The impact of regional industry-specific unionisation growth on residualised growth in industry-specific regional social security schemes conditional on region and industry time trends. First-difference linear IV-regressions.

	Uemployment	Disability, short-term	Disability, total	Disability, long-term	Retirement
**2. Step**					
ΔLagged lnU	-0.113**	-0.017**	-0.020**	-0.016**	0.024**
	(0.015)	(0.004)	(0.004)	(0.003)	(0.006)
ΔLagged lnL	0.148**	0.025**	0.028**	0.021**	-0.031**
	(0.022)	(0.005)	(0.006)	(0.004)	(0.009)
*Controls*					
Basic	Yes	Yes	Yes	Yes	Yes
Linear trends	Yes	Yes	Yes	Yes	Yes
**1. Step**					
$\tilde{\Delta U}$ (in 1000)	0.012**	0.012**	0.012**	0.012**	0.013**
	(0.001)	(0.001)	(0.001)	(0.001)	(0.001)
ΔLagged lnL	1.264**	1.264**	1.264**	1.264**	1.287**
	(0.041)	(0.041)	(0.041)	(0.041)	(0.041)
*Strength*					
F-value	105.129	105.129	105.129	105.129	61.843
MxIxT	15784	15784	15784	15784	13824

Panel unit: municipalityXindustry. Population yearly municipalityXindustry-sum and averages based on *all private sector* jobs. Dependent variable in: Δaverage regional industry-specific residuals of utilisation of social security schemes as indicated by column head (see A4 Table in S1 Appendix). Control vector: Basic = lagged municipality unemployment rate, year dummies; Linear trends = linear industry trends, linear municipality trends. Each observation is weighted by the number of workers. Standard errors adjusted for panel unit-clustering reported in parentheses.

** 1 percent level of significance

* 5 percent level of significance

$\Delta \Delta \tilde{\Delta U}\Delta$Table 6 reveals that increased unionisation implies reduced inflow to unemployment, and to disability in the short- and long-run. When unions raise the overall market wages causing the closure of the least productive firms and non-employment to increase, our results imply that laid-off workers do not stay on unemployment and on disability insurance. However, we do observe an increase in the inflow to retirement. However, the growth in non-employment not only relates to increased retirement, but although we do not observe participation in the educational system, non-employment might also reflect reskilling of workers laid-off. The presence of a strong local union might also contribute to local policies for reskilling of workers, thus limiting the entry to unemployment and disability.

## Conclusion

Our analyses reveal that local unionisation growth causes wage growth, both in unionised and non-unionised workplaces. On one hand, this wage growth then causes plants to close, and thus layoffs increase as well. On the other hand, hires following plant entry appear less sensitive to unionisation growth. Still, for some workers, this wage growth costly, since they are laid-off. Only the incumbent workers will benefit from the wage growth. However, most workers laid-off will find new work, since unemployment and disability rates remain largely unaffected, but some will chose retirement.

What about the employers? They will clearly have to pay higher wages. However, we also show that increased unionisation yields productivity gains. In this respect, our results support Barth et al. [6], which finds positive effects of firm union density on firm productivity. They suggest that this gain can be due to union voice effects or that when unions cross certain union density thresholds, they increasingly influence overall work organisation and policies at the firm level. The sum of such changes then yields the productivity gains. This productivity growth also benefits workers, thus Barth et al. [6] describe a rent-sharing mechanism. Our study also identify a rent-sharing mechanism. However, we find a strong productivity hike on average and at the top of the productivity distribution following increased local unionisation, while nothing happens at the bottom of the distribution. When wages, but not productivity, increase at the bottom of the wage distribution, this follows not from a rent-sharing mechanism. Similarly, a rent-sharing mechanism does not explain why the closure rates increase. On the other hand, if higher wages, due to unions, push less productive firms into closure, increased unionisation should increase closure rates. While the closure of less productive firms could contribute to higher productivity on average, it does not explain the increased productivity at the top.

Our interpretation is that unionisation contributes to local productivity growth through three channels. First, unionisation contributes to increased productivity at existing firms. Second, unionisation leaves the entry rate of new workplaces unaffected, but induces higher productivity among those that enters. Third, unionisation forces less productive workplaces to close due higher labour costs. These closures allocate resources to new and existing productive firms. In this respect, unions thereby contribute to creative destruction.

Given that unions are in decline, from a policy point of view, one implication from our study is that one should seek ways to bolster union membership and collective organisations. Another implication is that if such policies are successful, public policies need to address how to keep older workers from retiring.

## Supporting information

S1 Data (DOCX)

S1 Appendix (DOCX)
