# Supplementary material for: Do unions contribute to creative destruction?
Source: PLoS One. 2021 Dec 13;16(12):e0261212. doi: 10.1371/journal.pone.0261212 (PMC8668101; doi:10.1371/journal.pone.0261212)
Supplement: S1 Data — (DOCX) [file pone.0261212.s001.docx]

**Data availability statement**

Data used in this paper are mainly provided by Statistics Norway, but some are provided by the Norwegian Centre for Research Data through their Municipality Data Base (MDB). Statistics Norway provides microdata for research projects, and has data relating to persons, establishments and enterprises (for details, see Statistics Norway’s web page <https://www.ssb.no/en/omssb/tjenester-og-verktoy/data-til-forskning>). Data used in this paper are available to other researchers. However, note that transfer of personal data outside Norway’s borders is not allowed according to the statistics act. This means that any researcher or institution that wishes to apply for microdata will only ever be granted access to completely anonymous data. Due to the difficulty of anonymizing data in such a small country as Norway, this will rarely be feasible. Data used in our study is not anonymous.

The alternative approach is to gain access to a Norwegian research project and be given access at the Norwegian Institution in connection with such a project. This requires that the participating members be pre-submitted to Statistics Norway and that the formal requirements below are fulfilled. Institute for Social Research (Institutt for samfunnsforskning) is a major Norwegian research institution, approved by the Statistics Norway, and is usually running several projects utilising data from Statistics Norway. Institute for Social Research is welcoming collaboration with foreign researchers. Not all projects utilise the same data, and access to data is only ensured a limited amount of time. Thus, data used in this paper will not necessary always be available, but can be gained access to. Access to data from the Statistics Norway is unfortunately costly.

If you are a researcher at an approved research institution you can apply for access to data for a research project. If your institution is not on the list of approved research institutions, the institution must apply for approval to personvernombudet@ssb.no. If you are not associated with an approved research institution, you can also apply for access to data from Statistics Norway if your research institution is conducting a specific project that is either 1) financed by The Research Council of Norway or other national or international research program, or 2) on behalf of a public body that uses the Standard agreement for research and report assignments (the research agreement).

In addition, since the project will be processing personal data, you need a legal basis according to the General Data Protection Regulation (GDPR). Institute for Social Research has a Data Protection Officer to assist you in this process. The project needs a Data Protection Impact Assessment – DPIA.

Data used in this paper are from the standard list of data and variables presented at the Statistics Norway web page (https://www.ssb.no/en/omssb/tjenester-og-verktoy/data-til-forskning)Variables in the lists are electronically linked to available metadata (definitions and code lists). Your completed variable list must be attached to your application for research data. This paper utilise data from the following categories of data: Accounting data, Central Register of Establishments and Enterprises, Earnings, Education, Income, Labour market, Labour market and earnings, and Population.

The historical unionisation data used in this paper is provided by Norwegian Centre for Research Data through their Municipality Data Base (MDB). These data are available for Norwegian researchers at universities, colleges and research institutes upon application. The practice of making these data available to non-Norwegians outside Norway is not well-established. Contact information: kdb@nsd.no (MDB) or [kdb@nsd.no](mailto:kdb@nsd.no) (Norwegian Centre for Research Data)
