# Supplementary material for: Do unions contribute to creative destruction?
Source: PLoS One. 2021 Dec 13;16(12):e0261212. doi: 10.1371/journal.pone.0261212 (PMC8668101; doi:10.1371/journal.pone.0261212)
Supplement: S1 Appendix — (DOCX) [file pone.0261212.s002.docx]

**Appendix**

**Table A1. Descriptive statistics. Growth.**

|  | **Mean** | **Standard Deviation** |  | **Mean** | **Standard Deviation** |
| --- | --- | --- | --- | --- | --- |
| 1. All | | | | | |
| $\boldsymbol{\Delta}$LnW_t_ | 0.030 | 0.188 | $\Delta$Wage residual_t_ | 0.001 | 0.082 |
| $\boldsymbol{\Delta}$LnW_t_^Union^ | 0.026 | 0.385 | $\Delta$Wage residual_t_**^Union^** | -0.001 | 0.143 |
| $\boldsymbol{\Delta}$LnW_t_^Nonunion^ | 0.029 | 0.221 | $\Delta$Wage residual_t_**^Nonunion^** | 0.001 | 0.099 |
| $\boldsymbol{\Delta}$ lnU_t-1_ | 0.008 | 0.416 | $\Delta$Hires-Entry (HE) **_t_** | -0.001 | 0.086 |
| $\boldsymbol{\Delta}$ $\tilde{\boldsymbol{U}}$_t-1_ | 0.707 | 67.116 | $\Delta$HE residual**_t_** | -0.001 | 0.110 |
| $\boldsymbol{\Delta}$Ln employment_t-1_ | 0.007 | 0.241 | $\Delta$Layoffs-Exit (LE) **_t_** | 0.001 | 0.127 |
| $\boldsymbol{\Delta}$Unemployment_t_ | -0.002 | 0.098 | $\Delta$LE residual**_t_** | 0.002 | 0.114 |
| $\boldsymbol{\Delta}$Disability-short term_t_ | -0.001 | 0.038 | $\Delta$Hire-Increase(HI) **_t_** | -0.004 | 0.108 |
| $\boldsymbol{\Delta}$Disability-total_t_ | -0.001 | 0.046 | $\Delta$HI residual**_t_** | 0.001 | 0.107 |
| $\boldsymbol{\Delta}$Disability- t+3 _t_ | -0.001 | 0.034 | $\Delta$Quit-Decrease(QD) _t_ | 0.001 | 0.061 |
| $\boldsymbol{\Delta}$Retirement_t_ | -0.007 | 0.057 | $\Delta$QD-residual_t_ | -0.001 | 0.058 |
| $\boldsymbol{\Delta}$Retirement-residual _t_ | -0.001 | 0.046 | $\Delta$Disab.-short term-residual _t_ | 0.001 | 0.039 |
| $\boldsymbol{\Delta}$Unemploy-residual _t_ | 0.001 | 0.074 | $\Delta$Disab-total-residual _t_ | 0.001 | 0.045 |
|  |  |  | $\Delta$Disab.-residual - t+3 _t_ | 0.001 | 0.033 |
| MxIxT | 15284 |  |  | 15284 |  |
| 1. Panel units where TFP can be estimated | | | | | |
| $\boldsymbol{\Delta}$LnW_t_ | 0.025 | 0.133 | $\Delta$TFP_t_ | 0.028 | 0.241 |
| $\boldsymbol{\Delta}$Wage residual_t_ | 0.001 | 0.050 | $\Delta$95-5 TFP_t_ | 0.054 | 0.640 |
| $\boldsymbol{\Delta}$St.dev. wage residual_t_ | 0.004 | 0.064 | $\Delta$5^th^perc.. TFP_t_ | 0.001 | 0.313 |
| $\boldsymbol{\Delta}$5^th^perc.wage residual_t_ | -0.010 | 0.130 | $\Delta$95^th^perc. TFP_t_ | 0.055 | 0.561 |
| $\boldsymbol{\Delta}$95^th^perc.wage residual_t_ | 0.004 | 0.213 | $\Delta$ lnU_t-1_ | 4.155 | 1.745 |
| $\boldsymbol{\Delta}$LnC | 0.082 | 0.749 | $\Delta$ $\tilde{U}$_t-1_ | 112.051 | 1310.442 |
| $\boldsymbol{\Delta}$95-5 LnC _t_ | 0.151 | 1.882 | $\Delta$LnL | 0.043 | 0.493 |
| $\boldsymbol{\Delta}$5^th^perc. LnC _t_ | 0.009 | 1.254 | $\Delta$95-5 LnL _t_ | 0.132 | 1.255 |
| $\boldsymbol{\Delta}$95^th^perc. LnC _t_ | 0.160 | 1.448 | $\Delta$5^th^perc. LnL _t_ | -0.001 | 0.426 |
|  |  |  | $\Delta$95^th^perc. LnL _t_ | 0.131 | 1.190 |
| MxIxT | 10449 |  |  | 10449 |  |

Panel unit: municipalityXindustry. Panel A): Population: 15284 observations of yearly municipalityXindustry-sums and averages based on all private sector jobs 2003-2012. Note that the union- and non-union-specific figures rest on 14616 and 15218 observations, respectively. Panel B): 10449 observations on the municipalityXindustryXyear values from the estimated unobserved productivity from an auxiliary firm-level Translog production function estimation, and similar values for log fixed assets (LnC) and log workforce size(lnL).

**Table A2. Descriptive statistics on unionisation, predicted unionisation and employment. Across panel units.**

| Year | $\boldsymbol{\Delta}\tilde{\boldsymbol{U}}$<0 | | | No change | | | $\boldsymbol{\Delta}\tilde{\boldsymbol{U}}$>0 | | |
| --- | --- | --- | --- | --- | --- | --- | --- | --- | --- |
|  | Mean and standard deviation of yearly growth | | | | | | | | |
|  | $\Delta U$ | $\Delta\tilde{U}$ | $\Delta L$ | $\Delta U$ | $\Delta\tilde{U}$ | $\Delta L$ | $\Delta U$ | $\Delta\tilde{U}$ | $\Delta L$ |
| 2005 | -27 | -11 | -34 | -3 | 0 | 8 | 143 | 92 | 218 |
|  | (74) | (39) | (114) | (42) | (-) | (68) | (862) | (427) | (1178) |
| 2006 | -9 | -8 | 8 | -1 | 0 | 4 | 17 | 7 | 85 |
|  | (100) | (27) | (155) | (47) | (-) | (57) | (180) | (32) | (563) |
| 2007 | -81 | -18 | -105 | 4 | 0 | 4 | 35 | 26 | 66 |
|  | (166) | (43) | (222) | (29) | (-) | (48) | (196) | (153) | (365) |
| 2008 | 33 | -2 | 48 | 7 | 0 | 19 | 57 | 43 | 154 |
|  | (165) | (2) | (227) | (42) | (-) | (87) | (368) | (284) | (874) |
| 2009 | 7 | -8 | -4 | 5 | 0 | 11 | 58 | 52 | 131 |
|  | (118) | (33) | (242) | (37) | (-) | (66) | (637) | (304) | (1088) |
| 2010 | -42 | -23 | -81 | 3 | 0 | 5 | 251 | 250 | 243 |
|  | (253) | (99) | (520) | (78) | (-) | (109) | (1179) | 769) | (1166) |
| 2011 | -200 | -132 | -253 | -7 | 0 | -8 | 22 | 14 | 26 |
|  | (1333) | (667) | (1537) | (79) | (-) | (107) | (239) | (50) | (340) |
| 2012 | -11 | -8 | -2 | 1 | 0 | 6 | 11 | 15 | 118 |
|  | (104) | (32) | (205) | (29) | (-) | (63) | (133) | (76) | (608) |
| 1. Sums of yearly levels | | | | | | | | | |
|  | $\Sigma U$ | $\Sigma\tilde{U}$ | $\Sigma L$ | $\Sigma U$ | $\Sigma\tilde{U}$ | $\Sigma L$ | $\Sigma U$ | $\Sigma\tilde{U}$ | $\Sigma L$ |
| 2005 | 158047 | 90974 | 286871 | 216376 | 17 | 566830 | 129301 | 113141 | 372706 |
| 2006 | 152898 | 87625 | 281892 | 213915 | 35 | 573863 | 134152 | 115154 | 387304 |
| 2007 | 35930 | 23975 | 66053 | 220529 | 44 | 578374 | 256927 | 184649 | 615931 |
| 2008 | 4462 | 2309 | 9066 | 234882 | 198 | 61556 | 305547 | 218513 | 717720 |
| 2009 | 85603 | 42390 | 153495 | 244329 | 327 | 637572 | 236494 | 187812 | 598168 |
| 2010 | 134819 | 78713 | 246734 | 251352 | 115 | 647028 | 186436 | 155956 | 493183 |
| 2011 | 219963 | 169292 | 549915 | 237002 | 55 | 629651 | 76911 | 49382 | 158838 |
| 2012 | 111469 | 69919 | 207659 | 238739 | 124 | 641274 | 183837 | 148803 | 514405 |

$\tilde{U}$, and L denote lagged number of union workers, lagged predicted number of union worker and lagged total employment, respectively. $\Delta$ denotes the first-difference operator, while $\Sigma$ denotes aggregate sum. Due to the presence of lagged variables in the regression equation, 2005 is the first year of observation utilised in the regression. Panel unit: municipalityXindustry. Population: 15284 yearly municipalityXindustry-observations (averages, sums) based on *all private sector* jobs over the period 2005-2012. As denoted by column head, data is split into three categories depending on whether predicted unionisation declines, grows or show no changes. Panel A) presents descriptives (mean/standard deviation) on growth in lagged unionisation, growth in lagged predicted unionisation (our instrument) and lagged employment growth. Panel B) presents the yearly aggregate sums of lagged unionisation, lagged predicted unionisation and lagged employment.

**Table A3. Individual auxiliary regressions on wages, hires and layoffs. Private and public sector workers. 2003-2012. OLS regressions.**

|  | Log hourly wage | Hire due to plant entry | Hire due to plant entry | Layoff due to plant closure | Layoff due to workforce reductions |
| --- | --- | --- | --- | --- | --- |
| Woman | -0.180^**^(0.001) | -0.007^**^(0.001) | 0.007^**^(0.001) | -0.003^**^(0.001) | 0.001^**^(0.0001) |
| Immigrant | -0.080^**^(0.001) | 0.016^**^(0.001) | 0.009^**^(0.001) | 0.007^**^(0.001) | 0.003^**^(0.001) |
| Experience | 0.018^**^(0.001) | -0.001^**^(0.0001) | -0.008^**^(0.0001) | -0.001^**^(0.0001) | -0.003^**^(0.0001) |
| Experience^2^ | -0.0003^**^(0.0001) | 0.0001^**^(0.0001) | 0.001^**^(0.0001) | 0.0001^**^(0.00001) | 0.0001^**^(0.00001) |
| Seniority | 0.006^**^(0.001) | -0.019^**^(0.001) | -0.034^**^(0.001) | -0.003^**^(0.001) | 0.001^**^(0.0001) |
| Seniority^2^ | -0.0001^**^(0.0000) | 0.001^**^(0.00001) | 0.001^**^(0.0001) | 0.0001^**^(0.00001) | -0.000 1^**^(0.00001) |
|  | + all regressions comprise an intercept, year dummies, 6 dummies for educational qualifications, and dummies for 5-digit industry | | | | |
| JxPxT | 19,047,947 | 19,047,947 | 19,047,947 | 19,047,947 | 19,047,947 |

Panel unit: Job (or individual J’s employment relationship at workplace. Dependent variable denoted by column head. For hire and layoffs, the dependent variables are dummies.

^**^ 1 percent level of significance

^*^ 5 percent level of significance

**Table A4. Individual auxiliary regressions on social security uptake. Private and public sector workers. 2003-2012. OLS regressions.**

|  | Unemploy-ment | Disability short term | Disability total | Long term disability t+3 | Retirement |
| --- | --- | --- | --- | --- | --- |
| Woman | 0.0012^**^ (0.0001) | 0.0164^**^ (0.0001) | 0.0260^**^(0.0001) | 0.0143^**^ (0.0001) | 0.0153^**^(0.0001) |
| Immigrant | 0.0191^**^ (0.001) | -0.0067^**^(0.0001) | -0.0168^**^(0.0001) | -0.0137^**^(0.0001) | -0.0044^**^(0.0001) |
| Experience | 0.0008^**^ (0.001) | 0.0032^**^(0.0001) | -0.0008^**^(0.0001) | -0.0031^**^(0.0001) | -0.0153^**^(0.0001) |
| Experience^2^ | -0.0001^**^(0.0001) | -0.0001^**^(0.0001) | 0.0001^**^(0.00001) | 0.0001^**^(0.00001) | 0.0004^**^(0.00001) |
| Seniority | -0.0067^**^(0.0001) | -0.0019^**^(0.0001) | -0.0028^**^(0.0001) | -0.0001^**^(0.00001) | -0.0012^**^(0.0001) |
| Seniority^2^ | 0.0002^**^ (0.0001) | 0.0001^**^(0.00001) | 0.0001^**^(0.00001) | 0.0001^**^(0.00001) | 0.0001^**^ (0.00001) |
|  | + all regressions comprise an intercept, year dummies, 7 dummies for educational qualifications, and dummies for 5-digit industry | | | | |
| R^2^-adj. | 0.0516 | 0.0160 | 0.0541 | 0.0656 | 0.1505 |
| JxPxT | 19,214989 | 19,214989 | 19,214989 | 19,214989 | 19,214989 |

Panel unit: Job (or individual J’s employment relationship at workplace P). Dependent variable denoted by column head..

^**^ 1 percent level of significance

^*^ 5 percent level of significance

**Table A5. Firm auxiliary regression. Private sector firms. 2001-2012. GMM regressions.**

|  | | Log value added |
| --- | --- | --- |
| Ln workforce size | | 0.766^***^  (0.001) |
| Ln capital | | -0.001  (0.005) |
| Ln workforce size^2^ | | 0.046^**^  (0.001) |
| Ln capital^2^ | | 0.017^**^  (0.001) |
| Ln workforce sizeX Ln capital | | -0.005  (0.004) |
|  | + all regressions comprise an intercept, year dummies | |
| FxT | | 734343 |

Dependent variable: log value added. Capital is expressed by the fixed asset values. Productivity is estimated for each firm as unobserved TFP based on a Trans-log value added production function using standard two stage GMM-estimation (Ackerman, Caves, and Frazier [55], Rovigatti and Mollisi [58]) involving a proxy variable based on a third degree polynomial in intermediate materials, labour and the state variable capital (all variables in log form). Average log capital and log workforce size in these data are 6.5 and 1.6, respectively. The third quartile figures are 7.9 and 2.3, but the corresponding max values grow to close to 19 and 10. Firm-cluster adjusted standard errors based on 50 bootstrap replications.

^**^ 1 percent level of significance

^*^ 5 percent level of significance

**Table A6. The correlations between regional industry-specific unionisation growth on growth in industry-specific regional log hourly wage residuals. First-difference linear OLS- regressions.**

|  |  |  |  | Historically unionised municipalities only | Union | Non-union |
| --- | --- | --- | --- | --- | --- | --- |
|  | 1 | 2 | 3 | 4 | 5 | 6 |
| $\boldsymbol{\Delta}$Lagged lnU | 0.001 | 0.002 | 0.002 |  | -0.003 | 0.001 |
|  | (0.003) | (0.003) | (0.003) |  | (0.006) | (0.005) |
| $\boldsymbol{\Delta}$Lagged lnL | -0.001 | -0.002 |  |  | -0.002 | 0.001 |
|  | (0.005) | (0.005) |  |  | (0.009) | (0.003) |
| $\boldsymbol{\Delta}$Lagged lnL manufacturing |  |  | -0.002 |  |  |  |
|  |  |  | (0.005) |  |  |  |
| *Controls* |  |  |  |  |  | |
| Basic | Yes | Yes | Yes |  | Yes | Yes |
| Linear trends |  | Yes | Yes |  | Yes | Yes |
|  |  |  |  |  |  |  |
| MxIxT | 15284 | 15284 | 15284 |  | 14793 | 15218 |

Panel unit: municipalityXindustry. Population yearly municipalityXindustry-sum and averages based on *all private sector* jobs. Dependent variable (Y): log hourly wage residual (see Table A3). Control vector: Basic=lagged municipality unemployment rate, year dummies; Linear trends=linear industry trends, linear municipality trends. Each observation is weighted by the number of workers. Standard errors adjusted for panel unit-clustering reported in parentheses.

^**^ 1 percent level of significance

^*^ 5 percent level of significance

**Table A7. The correlations between regional unionisation growth on residualised job creation and destruction. First-difference linear OLS- regressions.**

|  | Hires | | Exit | | | |
| --- | --- | --- | --- | --- | --- | --- |
|  | Entry | Job creation | Job destruction | Exit | |  |
| $\boldsymbol{\Delta}$Lagged lnU | 0.001 | 0.025^**^ | 0.008^**^ | | 0.007 | |
|  | (0.005) | (0.008) | (0.002) | | (0.004) | |
| $\boldsymbol{\Delta}$Lagged lnL | -0.073^**^ | -0.165^**^ | -0.020^**^ | | -0.003 | |
|  | (0.010) | (0.025) | (0.005) | | (0.007) | |
| *Controls* |  |  |  | |  | |
| Basic | Yes | Yes | Yes | | Yes | |
| Linear trends | Yes | Yes | Yes | | Yes | |
| MxIxT | 15284 | 15284 | 15284 | | 15284 | |

Population yearly municipalityXindustry-sums and averages based on *all private sector* jobs. Dependent variable (Y): hires due to plant entry/hires in growing plants/separations in decreasing plants/layoffs due to plant closure (residuals, see Table A3). Control vector: Basic=lagged municipality unemployment rate, year dummies; Linear trends=linear industry trends, linear municipality trends. Standard errors adjusted for panel unit-clustering reported in parentheses.

^**^ 1 percent level of significance

^*^ 5 percent level of significance

**Table A8. The impact of regional industry-specific unionisation growth on residualised growth in industry-specific regional social security schemes conditional on region and industry time trends. First-difference linear OLS- regressions.**

|  | Uemploy-ment(t) | Disability, short(t) | Disability, total(t) | Disability, t+3 | Retire-ment(t) |
| --- | --- | --- | --- | --- | --- |
|  | 1 | 2 | 3 | 4 | 5 |
| $\boldsymbol{\Delta}$Lagged lnU | -0.005^*^ | 0.002 | 0.001 | 0.001 | -0.001 |
|  | (0.002) | (0.001) | (0.001) | (0.001) | (0.001) |
| $\boldsymbol{\Delta}$Lagged lnL | 0.009^*^ | 0.001 | 0.001 | 0.001 | -0.001 |
|  | (0.004) | (0.001) | (0.001) | (0.001) | (0.003) |
| *Controls* |  |  |  | | |
| Basic | Yes | Yes | Yes | Yes | Yes |
| Linear trends | Yes | Yes | Yes | Yes | Yes |
|  |  |  |  | |  |
| MxIxT | 15784 | 15784 | 15784 | 15784 | 13824 |

Panel unit: municipalityXindustry. Population yearly municipalityXindustry-sum and averages based on *all private sector* jobs. Dependent variable: Δaverage regional industry-specific residuals of utilisation of social security schemes as indicated by column head (see Table A4). Control vector: Basic=lagged municipality unemployment rate, year dummies; Linear trends=linear industry trends, linear municipality trends. Each observation is weighted by the number of workers. Standard errors adjusted for panel unit-clustering reported in parentheses.

^**^ 1 percent level of significance

^*^ 5 percent level of significance

**Table A9. Correlation historic union industry-shares and 2003-labour supply characteristics. OLS regressions.**

|  | Model 1 | Model 2 | Model 3 |
| --- | --- | --- | --- |
| Women | 0.016 (0.016) | -0.036 (0.034) | -0.042 (0.034) |
| Immigrants | 0.133 (0.116) | 0.093 (0.088) | 0.067 (0.086) |
| Education (years of) | 0.303 (0.189) | 0.188 (0.122) | 0.154 (0.122) |
| Experience | 0.216 (0.173) | 0.245 (0.177) | 0.285 (0.187) |
| Seniority | -0.002 (0.023) | -0.020 (0.032) | -0.066 (0.048) |
| Age | -0.178 (0.123) | -0.240(0.144) | -0.184(0.124) |
| Log workers 2003 |  |  | 0.310^*^ (0.138) |
| Log workers 1920 |  | 0.383^*^(0.184) |  |
| M | 415 | 415 | 415 |

This table reports the results from a OLS regression of the first principal component of 1918 union industry shares on 2003-labour supply characteristics. Unit of observation: municipality, denoted by M. Dependent variable: first principal component of 1918 union industry shares. Each characteristic is standardized to have unit standard deviation. The first principal component also has unit standard deviation. Standard errors in parentheses.

^**^ 1 percent level of significance

^*^ 5 percent level of significance

**Table A10. The short- and long-run impact of regional unionisation growth on growth in regional log hourly wages. First-difference linear IV-regressions.**

|  | 1^st^ stage | |  | 2^nd^ stage |
| --- | --- | --- | --- | --- |
|  | $\Delta$lnU_t-1_ | $\Delta$lnU_t-2_ | | $\Delta$Wage residual_t_ |
| $\boldsymbol{\Delta}$lnU_t-1_ |  |  | | 0.103^**^(0.015) |
| $\boldsymbol{\Delta}$lnU_t-2_ |  |  | | 0.015 (0.009) |
| $\boldsymbol{\Delta}$Lagged lnL | 1.287^**^(0.051) | -0.152^**^(0.046) | | -0.132^**^ (0.021) |
| ${\tilde{\boldsymbol{\Delta U}}}_{\mathbf{t-1}}$ | 1.3e-5^**^ (1.1e-6) | -5.1e-7 (1.1e-6) | |  |
| ${\boldsymbol{\Delta}\tilde{\mathbf{U}}}_{\mathbf{t-2}}$ | 8.3e-7^**^(4.0e-7) | 2.9e-5^**^(2.1e-6) | |  |
| *Controls* |  |  | |  |
| Basic | Yes | Yes | | Yes |
| Linear trends | Yes | Yes | | Yes |
| F-value excl.instruments | 121.62 | 259.99 | |  |
| K-P F-value |  |  | | 58.82 |
| MxIxT | 13102 | 13102 | | 13102 |

Panel unit: municipalityXindustry. Population: yearly municipalityXindustry-averages based on all private sector jobs. Dependent variable second stage: log hourly wage (residual). Control vector: Basic=lagged municipality unemployment rate, year dummies; Linear trends=linear industry trends, linear municipality trends. Standard errors adjusted for panel unit-clustering reported in parentheses.

^**^ 1 percent level of significance

^*^ 5 percent level of significance

**Table A11. Over-identification and monotonicity. The impact of the principal component of the historical municipality industry shares on growth in regional log hourly wages.**

**First-difference linear IV-regressions.**

|  | 1^st^ stage | 2^nd^ stage |
| --- | --- | --- |
| Dep.variable: | $\Delta$lnU_t-1_ | $\Delta$Wage residual_t_ |
| $\boldsymbol{\Delta}$lnU_t-1_ |  | 0.044^**^ (0.016) |
| $\boldsymbol{\Delta}$Hist.unionshare PC1X2005 | 0.285^**^ (0.030) |  |
| $\boldsymbol{\Delta}$Hist.unionshare PC1X2006 | 0.328^**^ (0.047) |  |
| $\boldsymbol{\Delta}$Hist.unionshare PC1X2007 | 0.229^**^ (0.066) |  |
| $\boldsymbol{\Delta}$Hist.unionshare PC1X2008 | 0.242^**^ (0.086) |  |
| $\boldsymbol{\Delta}$Hist.unionshare PC1X2009 | 0.378^**^ (0.109) |  |
| $\boldsymbol{\Delta}$Hist.unionshare PC1X2010 | 0.512^**^ (0.136) |  |
| $\boldsymbol{\Delta}$Hist.unionshare PC1X2011 | 0.186 (0.146) |  |
| $\boldsymbol{\Delta}$Hist.unionshare PC1X2012 | 0.129 (0.170) |  |
| $\boldsymbol{\Delta}$Lagged lnL | 1.289^**^ (0.002) | -0.057^**^ (0.022) |
|  |  |  |
| Controls |  |  |
| Basic | Yes |  |
| Linear trends | Yes |  |
| *Strength/Overidentification* |  |  |
| K-P F-value | 50.581 |  |
| Hansen P-value | 0.952 |  |
|  |  |  |
| MxIxT | 15284 | 15284 |

Panel unit: municipalityXindustry. Population yearly municipalityXindustry-averages based on all private sector jobs. Dependent variable second stage: log hourly wage (residual). Control vector: Basic=lagged municipality unemployment rate, year dummies; Linear trends=linear industry trends, linear municipality trends. Note the instrument vector comprises the first principal component of 1918 union industry shares across municipalities is interacted with year dummies. Since the first principal component vary only across municipalities, the reported standard errors (in parentheses) are adjusted for municipality-clustering.

^**^ 1 percent level of significance

^*^ 5 percent level of significance
